# Severe Morbidity and Mortality Associated With Respiratory Syncytial Virus Versus Influenza Infection in Hospitalized Older Adults

**DOI:** 10.1093/cid/ciy991

**Published:** 2018-11-19

**Authors:** Bradley Ackerson, Hung Fu Tseng, Lina S Sy, Zendi Solano, Jeff Slezak, Yi Luo, Christine A Fischetti, Vivek Shinde

**Affiliations:** 1 Department of Research and Evaluation, Kaiser Permanente Southern California, Pasadena; 2 Clinical Development, Novavax Inc., Gaithersburg, Maryland

**Keywords:** respiratory syncytial virus, influenza, adult, hospitalization, mortality

## Abstract

**Background:**

Respiratory syncytial virus (RSV) is an important cause of serious respiratory illness in older adults. Comparison of RSV and influenza infection in hospitalized older adults may increase awareness of adult RSV disease burden.

**Methods:**

Hospitalized adults aged ≥60 years who tested positive for RSV or influenza between 1 January 2011 and 30 June 2015 were identified from Kaiser Permanente Southern California electronic medical records. Baseline characteristics, comorbidities, utilization, and outcomes were compared.

**Results:**

The study included 645 RSV- and 1878 influenza-infected hospitalized adults. Patients with RSV were older than those with influenza (mean, 78.5 vs 77.4 years; *P* = .035) and more likely to have congestive heart failure (35.3% vs 24.5%; *P* < .001) and chronic obstructive pulmonary disease (COPD) (29.8% vs 24.3%; *P* = .006) at baseline. In adjusted analyses, RSV infection was associated with greater odds of length of stay ≥7 days (odds ratio [OR] = 1.5; 95% confidence interval [CI], 1.2–1.8; *P* < .001); pneumonia (OR = 2.7; 95% CI, 2.2–3.2; *P* < .001); intensive care unit admission (OR = 1.3; 95% CI, 1.0–1.7; *P* = .023); exacerbation of COPD (OR = 1.7; 95% CI, 1.3–2.4; *P* = .001); and greater mortality within 1 year of admission (OR = 1.3; 95% CI, 1.0–1.6; *P* = .019).

**Conclusions:**

RSV infection may result in greater morbidity and mortality among older hospitalized adults than influenza. Increased recognition of adult RSV disease burden will be important in the evaluation and use of new RSV vaccines and antivirals.

## Teaser

Older adults hospitalized with respiratory syncytial virus (RSV) or influenza were compared. RSV infection was associated with longer hospitalization and greater odds of pneumonia, intensive care unit admission, and 1-year mortality. Recognition of adult RSV disease is needed to facilitate future vaccination and treatment.

**(See the Editorial Commentary by Branche on pages 204–6.)**

Respiratory syncytial virus (RSV) initially was found to be a common cause of severe respiratory illness in young children [1, 2]. While mild disease in healthy young adults has also been described [3], the potential for RSV to cause severe disease in adults was not recognized until reports of outbreaks among older adults residing in long-term care facilities emerged [4, 5]. Subsequently, RSV was shown to cause serious illness among healthy, community-dwelling older adults. A landmark study of high-risk and community-dwelling adults aged ≥65 years and high-risk adults in the United States detected RSV infection in 3%–7% of healthy, community-dwelling older adults, 4%–10% of high-risk adults, and 16% of adults hospitalized with cardiopulmonary infections [6]. Furthermore, RSV infection was estimated to result in approximately 177000 hospitalizations, 10000 to 14000 deaths, and more than $1 billion dollars in healthcare costs per year in the United States, mostly among older adults [6, 7]. Nonetheless, RSV remains clinically underrecognized by most healthcare providers as a cause of severe respiratory disease in adults [7–9].

A current comparison of the clinical characteristics and burden of RSV illness with those of influenza, a well-recognized cause of severe morbidity and mortality in older adults [10], may increase provider awareness of the potential of RSV infection to cause severe lower respiratory tract disease in older adults. Appreciation of the potential for RSV infection to cause serious illness similar to that caused by influenza will likely trigger more testing for RSV in addition to testing for influenza and direct therapy to the identified etiology in patients as newer agents become available. In addition, recent data on the epidemiology and clinical outcomes of older adults hospitalized with RSV are critical to the evaluation of more than 50 RSV vaccines, monoclonal antibodies, and antivirals currently under development prior to their introduction [11]. While a number of important studies have described the burden and clinical characteristics of RSV and influenza disease in hospitalized older adults, most of these studies were conducted at least a decade ago [6, 12–15]. Furthermore, long-term mortality has not been analyzed previously. Therefore, we evaluated a large cohort of adults aged ≥60 years hospitalized with RSV or influenza A/B infection during 5 consecutive seasons. Demographics, prehospitalization characteristics, hospital utilization, and clinical outcomes, including long-term mortality, were assessed and compared between adults with RSV infection and those with influenza virus infection.

## METHODS

### Setting

This observational, retrospective, cohort study was conducted at Kaiser Permanente Southern California (KPSC), an integrated healthcare system that provides comprehensive prepaid health services for 4.4 million members cared for at 15 large, community-based hospitals that are owned and operated by KPSC. The demographic makeup of the KPSC membership closely mirrors the racially and sociodemographically diverse Southern California population and the California census population; more than 99% are community dwelling [16]. As KPSC is a prepaid healthcare system, there is a very strong motivation for members to use services internally. Furthermore, reimbursement by KPSC for outside care requires that claims be submitted with documentation of the care provided; claims are subsequently entered into the administrative data systems. Thus, capture of care delivered to KPSC members by electronic data is very comprehensive.

### Study Population

The study population consisted of hospitalized KPSC members aged ≥60 years at the time of admission who had a positive RSV or influenza A/B laboratory test (multiplex polymerase chain reaction [PCR] [FilmArray, Luminex xTAG], influenza A/B/H1N1 [Cepheid Xpert Flu], or viral culture) that was ordered between 1 January 2011 and 30 June 2015 during a hospitalization encounter or an emergency department encounter that resulted in hospitalization at 1 of 15 KPSC hospitals. All samples were submitted to a single, centralized KPSC laboratory for processing. Participants were required to have continuous KPSC membership for at least 6 months (allowing a 31-day gap) prior to the hospital admission date to allow for ascertainment of baseline characteristics. If there were patients with multiple hospitalizations who met the above criteria (eg, had a positive RSV or influenza A/B test on 2 separate hospitalization encounters), the first RSV or first influenza hospitalization was kept for the RSV or influenza cohort, respectively. For the purpose of statistical comparison, independent samples of RSV and influenza cohorts were defined. Twenty-five individuals with both a positive RSV test and a positive influenza test during different hospitalizations during the study period were kept in the RSV cohort and removed from the influenza cohort. Nineteen individuals coinfected with RSV and influenza during the same hospitalization were excluded from the comparison. The KPSC Institutional Review Board reviewed and approved the study.

### Source of Data

Electronic medical records were used to obtain information for the study population, including membership history, baseline characteristics, demographics, comorbidities, treatments, utilization, and outcomes. Each KPSC health plan member has a unique medical record number that is used as an identifier to retrieve and link all variables from different databases.

### Hospitalization Outcomes

Outcomes of hospitalization included length of stay, occurrence of complications, use of respiratory support, intensive care unit (ICU) admission, readmission, use of vasopressors, mortality, exacerbation of chronic conditions, discharge location, and healthcare utilization after discharge.

### Statistical Analyses

Patient demographics, prehospital course, comorbid conditions, and in-hospital utilization were compared between patients hospitalized with RSV and those hospitalized with influenza A/B using a χ^2^ test, Fisher exact test, *t* test, or Wilcoxon rank-sum test, as appropriate. Categorical factors were presented using frequencies and percentages. Continuous variables were categorized based on distribution and described using mean, standard deviation, and range, in addition to frequencies and percentages.

Multivariable logistic regression was used to estimate the odds ratio (OR) and 95% confidence interval (CI) associated with hospitalization outcomes between those infected with RSV and those infected with influenza after adjustment for potential differences between cohorts, including age, sex, race/ethnicity, history of influenza and pneumococcal vaccination, prior chronic obstructive pulmonary disease (COPD)/chronic bronchitis/emphysema, Charlson comorbidity index [17], history of healthcare utilization, and recent use of antivirals, antibiotics, or steroids. One-year survival after admission was estimated using the Kaplan-Meier method, and survival rates between hospitalized persons infected with RSV or influenza were compared using the log-rank test. The analyses were performed using SAS Enterprise Guide 5.1 (SAS Institute Inc, Cary, NC).

## RESULTS

Among patients hospitalized with RSV (n = 645) during the study period, RSV infection was detected by multiplex PCR in 91.8% and by culture in 8.2% of patients. Among patients hospitalized with influenza (n = 1878) during the study period, influenza infection was detected by multiplex PCR in 82.6%, by influenza A/B/H1N1 PCR in 0.05%, and by culture in 17.4% of patients. The distribution of demographic characteristics, medical history, and comorbidities of patients hospitalized with RSV (n = 645) or with influenza (n = 1878) is presented in Table 1. Patients hospitalized with RSV infection were slightly older (mean age, 78.5 vs 77.4 years; *P* = .035), with a higher proportion of those aged ≥85 years compared to those hospitalized with influenza infection. There was a higher proportion of females in the RSV cohort than in the influenza cohort (60.5% vs 50.3% female; *P* < .001). Race/ethnicity distributions were similar between the RSV and the influenza cohorts. A higher proportion of RSV patients than influenza patients had received influenza vaccine in the 1 year prior to admission (83.1% vs 73.1%; *P* < .001). Smoking status and body mass index were similar between cohorts. The prevalence of several comorbidities was significantly different between patients hospitalized with RSV and those with influenza, including congestive heart failure (35.3% vs 24.5%; *P* < .001), diabetes (38.9% vs 44.6%; *P* = .012), COPD/chronic bronchitis/emphysema (29.8% vs 24.3%; *P* = .006), asthma (26.0% vs 18.6%; *P* < .001), any solid cancer (11.2% vs 8.0%; *P* = .016), and leukemia (1.9% vs 0.9%; P = .050). Use of any antiviral therapy, including oseltamivir, within 14 days prior to admission was not common but was more prevalent in the RSV cohort than the influenza cohort (4.3% vs 2.7%; *P* = .034 for any antiviral medication and 2.3% vs 1.1%; *P* = .026 for oseltamivir; data not shown).

**Table 1.** Baseline Characteristics of Patients Aged ≥60 Years Hospitalized With Respiratory Syncytial Virus or Influenza Infection in Kaiser Permanente Southern California, 2011–2015

Patient Characteristic	Respiratory Syncytial Virus (N = 645)	Influenza (N = 1878)	*P* Value^a^
Age (years) at admission (mean, SD, range)	78.5, 9.8, 60.0–103.0	77.4, 9.5, 60.0–104.0	.035
60–64 (%)	56 (8.7)	215 (11.4)	.039
65–74 (%)	176 (27.3)	506 (26.9)	…
75–84 (%)	220 (34.1)	685 (36.5)	…
85+ (%)	193 (29.9)	472 (25.1)	…
Sex			<.001
Male (%)	255 (39.5)	933 (49.7)	…
Female (%)	390 (60.5)	945 (50.3)	…
Race			.056
White (%)	425 (65.9)	1218 (64.9)	…
Black (%)	110 (17.0)	264 (14.1)	…
Asian/Pacific Islander (%)	63 (9.8)	245 (13.0)	…
Other/multiple/unknown (%)	47 (7.3)	151 (8.0)	…
Ethnicity			.578
Hispanic (%)	149 (23.1)	471 (25.1)	…
Non-Hispanic (%)	495 (76.7)	1405 (74.8)	…
Influenza vaccination in 1 year prior to admission (%)	536 (83.1)	1372 (73.1)	<.001
Pneumococcal vaccination in 5 years prior to admission (%)	313 (48.5)	831 (44.2)	.060
Smoking			.982
Never (%)	403 (62.5)	1175 (62.6)	..
Ever (%)	242 (37.5)	703 (37.4)	…
Body mass index (kg/m2)			.163
<18.5 (%)	26 (4.0)	83 (4.4)	…
18.5–24.99 (%)	241 (37.4)	635 (33.8)	…
25–29.99 (%)	195 (30.2)	631 (33.6)	…
30–39.99 (%)	142 (22.1)	446 (23.8)	…
40+ (%)	40 (6.2)	82 (4.4)	…
Comorbidities in 1 year prior to admission			
Ischemic heart disease (%)	208 (32.2)	569 (30.3)	.355
Congestive heart failure (%)	228 (35.3)	461 (24.5)	<.001
Diabetes (%)	251 (38.9)	837 (44.6)	.012
Chronic obstructive pulmonary disease, chronic bronchitis, or emphysema (%)	192 (29.8)	456 (24.3)	.006
Asthma (%)	168 (26.0)	349 (18.6)	<.001
End stage renal disease (%)	36 (5.6)	117 (6.2)	.552
Any solid cancer (%)	72 (11.2)	151 (8.0)	.016
Leukemia (%)	12 (1.9)	17 (0.9)	.050
Lymphoma (%)	14 (2.2)	32 (1.7)	.445
Prehospital utilization			
Any antiviral drug use in 14 days prior to admission (%)	28 (4.3)	50 (2.7)	.034
Duration of antiviral drug use in 14 days prior to admission, days (mean, SD range)	8.0, 6.0, 1.0–15.0	9.0, 5.9, 1.0–15.0	.322

Abbreviation: SD, standard deviation.

^a^ *P* value from χ^2^ test, *t* test, Fisher exact test, or Wilcoxon rank-sum test, as appropriate. Unknown ethnicity and unknown body mass index are eliminated.

The in-hospital characteristics of the RSV and influenza cohorts are described in Table 2. The mean time from admission to the first positive test result for either RSV or influenza was similar between the 2 cohorts (2.5 vs 2.6 days; *P* = .570), and approximately 98% of patients in either cohort did not have test results available until some time after the hospital admission date. Among the RSV and influenza cohorts, respectively, use of antivirals (47.1% vs 78.6%; *P* < .001), 99% of which was oseltamivir (data not shown); antibiotics (94.1% vs 88.9%; *P* < .001); and any inhalation, injection, intravenous, or oral steroid (64.5% vs 47.9%; *P* < .001) during the hospitalization period was significantly different.

**Table 2.** In-hospital Characteristics of Patients Aged ≥60 Years Hospitalized With Respiratory Syncytial Virus or Influenza Infection in Kaiser Permanente Southern California, 2011–2015

Patient Characteristics	Respiratory Syncytial Virus (N = 645)	Influenza (N = 1878)	*P* Value^a^
Days from admission to positive RSV/influenza test order date (mean, SD, range)	0.7, 1.9, 0.0–31.0	0.7, 1.4, 0.0–20.0	.402
Days from admission to positive RSV/influenza test result date (mean, SD, range)	2.5, 2.6, 0.0–35.0	2.6, 1.9, 0.0–21.0	.570
Any antiviral drug use during hospitalization encounter (%)	304 (47.1)	1477 (78.6)	<.001
Any antibiotic drug use during hospitalization encounter (%)	607 (94.1)	1670 (88.9)	<.001
Any inhalation, injection, IV, or oral steroid use during hospitalization encounter (%)	416 (64.5)	900 (47.9)	<.001
Inhalation steroid use during hospitalization encounter (%)	183 (28.4)	418 (22.3)	.002
Injection steroid use during hospitalization encounter (%)	321 (49.8)	622 (33.1)	<.001
IV steroid use during hospitalization encounter (%)	6 (0.9)	12 (0.6)	.448
Oral steroid use during hospitalization encounter (%)	267 (41.4)	480 (25.6)	<.001
Respiratory co-infection within ±7 days of test order (%)^b^	9 (1.4)	20 (1.1)	.497

Abbreviations: IV, intravenous; RSV, respiratory syncytial virus; SD, standard deviation.

^a^ *P* value from χ^2^ test, *t* test, Fisher exact test, or Wilcoxon rank-sum test, as appropriate.

^b^Respiratory coinfections within ±7 days included parainfluenza, human metapneumovirus, rhinovirus/enterovirus, and coronavirus. Also, 1 person with c-infection of RSV and influenza within ±7 days but not during the same hospitalization was counted in the RSV cohort and removed from the influenza cohort.

**Figure 1.**
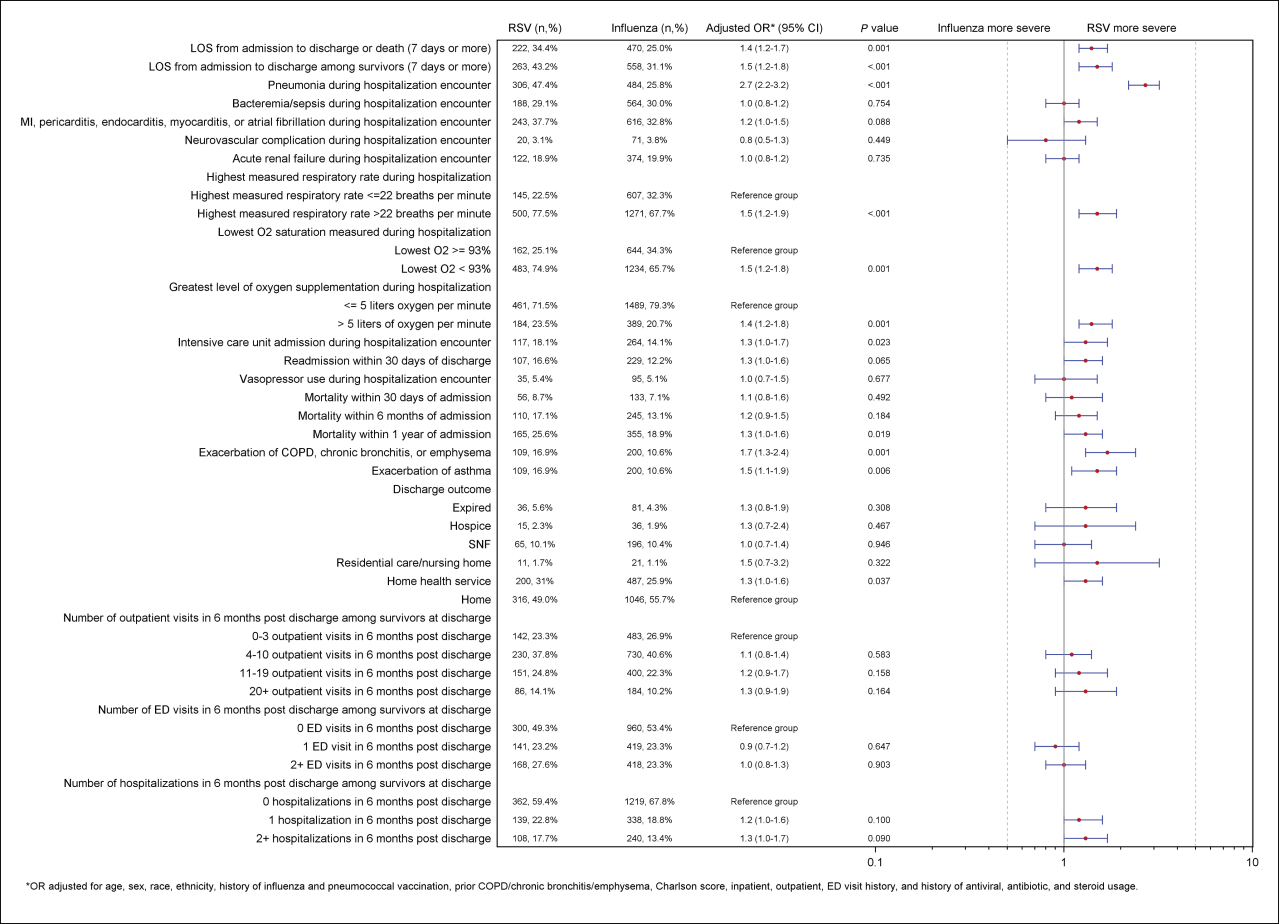
Comparison of selected outcomes among patients aged ≥60 years hospitalized with respiratory syncytial virus or influenza infection in Kaiser Permanente Southern California, 2011–2015. Abbreviations: CI, confidence interval; COPD, chronic obstructive pulmonary disease; ED, emergency department; LOS, length of stay; MI, myocardial infarction; OR, odds ratio; RSV, respiratory syncytial virus; SNF, skilled nursing facility.

**Figure 2.**
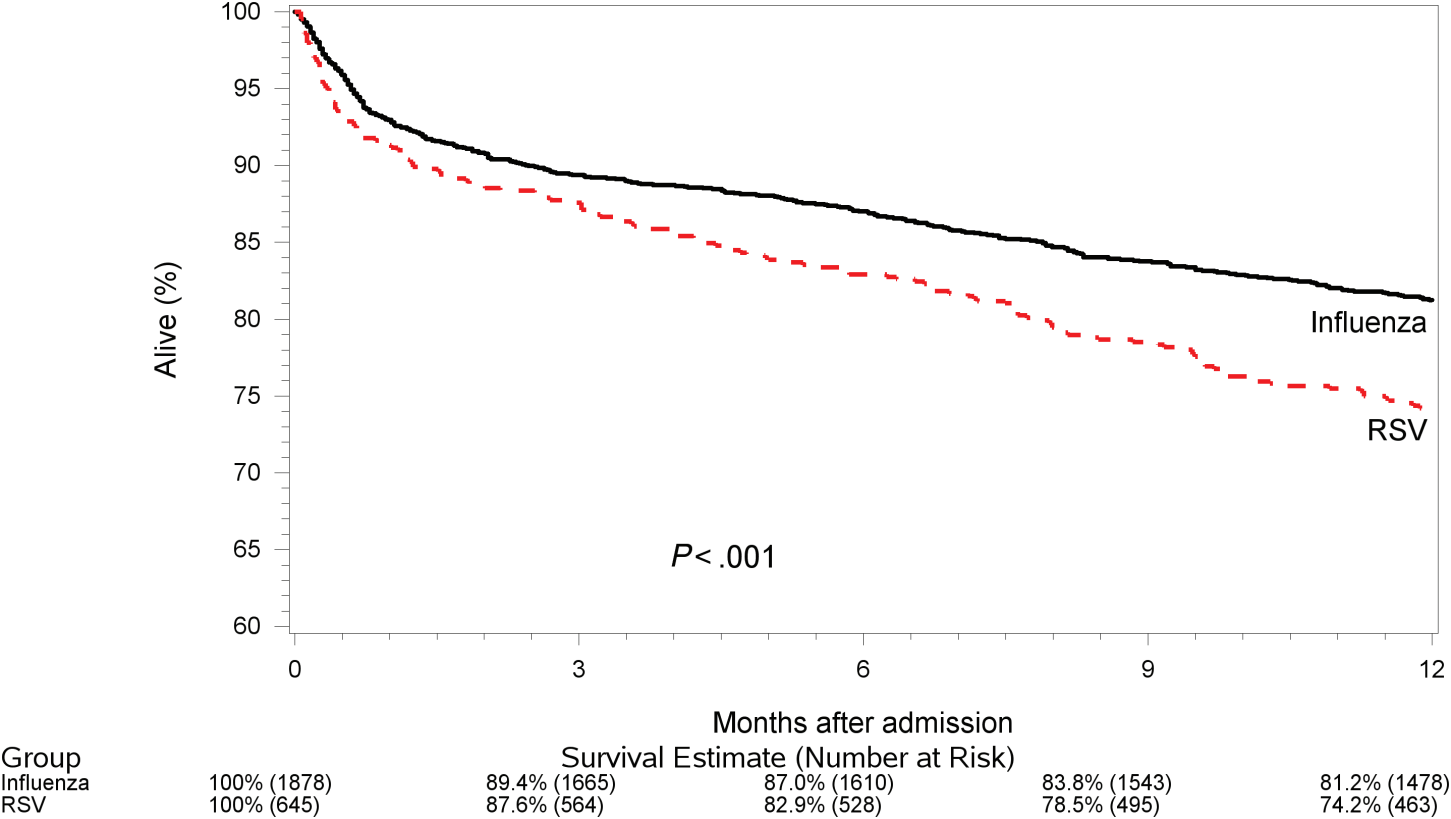
Rate of survival within 1 year of admission among patients aged ≥60 years hospitalized with respiratory syncytial virus or influenza infection in Kaiser Permanente Southern California, 2011–2015. Abbreviation: RSV, respiratory syncytial virus.

In the adjusted analyses, the odds of all outcomes were similar or significantly higher among participants hospitalized with RSV compared to those hospitalized with influenza (Figure 1). Hospital utilization was greater among those infected with RSV than with influenza as measured by length of stay ≥7 days among the entire population (OR = 1.4; 95% CI, 1.2–1.7), length of stay ≥7 days among survivors (OR = 1.5; 95% CI, 1.2–1.8), and ICU admission (OR = 1.3; 95% CI, 1.0–1.7). In addition, respiratory complications during hospitalization were more common among RSV-infected older adults including pneumonia diagnosis (OR = 2.7; 95% CI, 2.2–3.2); highest measured respiratory rate (RR; measured by breaths per minute; RR ≥26 vs ≤22 reference group [OR = 1.5; 95% CI, 1.2–1.9]); lowest oxygen saturation (≤84% vs ≥93% reference group [OR = 1.6; 95% CI, 1.2–2.1]); greatest level of oxygen supplementation (≥16 L/min vs ≤5 L/min reference group [OR = 1.5; 95% CI, 1.0–2.2]); exacerbation of COPD, chronic bronchitis, or emphysema (OR = 1.7; 95% CI, 1.3–2.4); and exacerbation of asthma (OR = 1.5; 95% CI, 1.1–1.9). More older adults infected with RSV than with influenza required home health service after discharge (OR = 1.3; 95% CI, 1.0–1.6). While mortality during hospitalization (OR = 1.1; 95% CI, 0.8–1.7) and within 6 months of admission (OR = 1.2; 95% CI, 0.9–1.5) for patients with RSV did not differ significantly from those with influenza, long-term mortality within 1 year of admission was significantly greater in the RSV cohort compared to the influenza cohort (OR = 1.3, 95% CI, 1.0–1.6). The 1-year survival rate after admission was significantly lower (Figure 2, 74.2% vs 81.2%; *P* < .001) in older adults hospitalized with RSV compared to those hospitalized with influenza.

## DISCUSSION

This retrospective cohort study that spans 5 consecutive winters demonstrates that RSV is an important cause of serious and life-threatening respiratory illness that results in morbidity and mortality among older adults that were comparable or more severe than those caused by influenza. To our knowledge, this study describes the largest and most recent cohort of older adults hospitalized with RSV or influenza reported to date. By utilizing patient electronic health records, we were able to obtain important information, including demographics and comorbidities prior to hospitalization and short- and long-term healthcare utilization and hospitalization outcomes.

Consistent with earlier reports, we found that adults hospitalized with RSV were slightly older and had greater frequency of baseline comorbidities, particularly cardiopulmonary disease, than those hospitalized with influenza virus infection [6, 12, 14]. However, after adjustment for comorbidities, we found that hospitalization outcomes among older adults hospitalized with RSV were either comparable to or significantly worse than they were among those hospitalized with influenza. While previous studies found similar hospital length of stay among older adults hospitalized with RSV or with influenza [6, 14], we found that longer hospitalizations (length of stay ≥7 days) occurred more often among the RSV cohort than they did in the influenza cohort. This may reflect increased use of antiviral therapies in recent years that ameliorate influenza but not RSV disease [18] or differences in clinical recovery associated with a more pronounced RSV-associated severe lower respiratory tract syndrome. We also found that pulmonary complications and findings, including pneumonia, tachypnea, hypoxia, and greater need for oxygen supplementation, and exacerbation of asthma and of COPD, chronic bronchitis or emphysema were significantly greater among the RSV cohort after adjustment for potential confounders. These findings are similar to those of Lee et al. who reported that hospitalized adults with RSV in Hong Kong had a greater frequency of lower respiratory complications than those with influenza [14]. While increased baseline cardiopulmonary disease in the RSV cohort likely increased their risk of pulmonary complications, including exacerbation of chronic respiratory disease and asthma, these differences were observed after adjustment for comorbidities, including baseline cardiopulmonary disease. Discharge to hospice and skilled nursing facilities was similar in the RSV and influenza cohorts, but a greater proportion of the RSV cohort required home health services after discharge. Finally, similar to previous findings [6, 14], short-term mortality for patients infected with RSV was similar to that of patients infected with influenza. However, longer-term survival among the RSV cohort, which has not been reported previously, was significantly worse than that of the influenza cohort. The impact of RSV on long-term survival may be a reflection of the potential for RSV to drive chronic inflammation associated with persistent, sometimes progressive, pulmonary disease, although the mechanism for this is not clear [19–22]. In addition to confirming the severe impact of annual influenza epidemics, our findings also underscore the substantial morbidity, mortality, and healthcare utilization associated with RSV infection in the expanding population of older adults and the need for RSV vaccines and antivirals.

Interestingly, influenza vaccine uptake within 1 year of admission was higher among the RSV cohort than the influenza cohort while receipt of pneumococcal vaccine was similar. This may reflect, in part, the effectiveness of influenza vaccine in preventing influenza infections and is unlikely due to differential vaccine acceptance. On the other hand, the increased use of oseltamivir within 14 days prior to hospitalization among older adults hospitalized with RSV compared to those hospitalized with influenza may reflect presumed influenza infection and delay in diagnosis of RSV infection. Conversely, decreased in-hospital use of antiviral therapy among the RSV cohort compared to the influenza cohort likely reflects the discontinuation of oseltamivir after identification of RSV and initiation of oseltamivir after identification of influenza. The greater use of antibiotics and systemic and inhaled steroids in the RSV cohort during their hospitalizations may mirror the higher frequency of pneumonia and exacerbation of chronic lung disease and asthma among older hospitalized adults with RSV infection than those with influenza infection.

Whereas RSV has long been recognized as a cause of serious illness among young children, its role as a significant pathogen among older adult patients appears to be underrecognized by many adult providers [7–9]. This is understandable since in many settings, respiratory pathogen testing is limited to influenza virus testing, so that noninfluenza pathogens, including RSV, have not been recognized as possible etiologies of serious respiratory illness similar to that caused by influenza. In addition, even in settings in which multiplex pathogen testing is used, local circulation of influenza virus in the community and clinical suspicion for influenza may drive testing patterns among hospitalized older adults and, thus, may underestimate the relative frequency of other viral respiratory pathogens, including RSV, which may have either nonoverlapping or broader periods of circulation in the same community. Therefore, the number of hospitalizations with RSV or with influenza detected in this study may not reflect the relative incidences of each pathogen over the study period since RSV infections that occur during intervals when influenza virus is less prevalent are less likely to be detected.

Increased provider recognition of RSV as an important cause of severe influenza-like illness among older adults and other high-risk populations will become increasingly important in order to increase vaccine uptake among high-risk populations after RSV vaccines are developed. In addition, recognition and early identification of RSV as a potential cause of illness that is sometimes indistinguishable from influenza [23, 24] will be important in the timely implementation of diagnostic testing, adjustment of antiviral medication administration, including early discontinuation of medications targeted to influenza, and initiation of medications effective against RSV as they become available in order to optimize effectiveness and to minimize cost and complications.

The strengths of this study include detailed clinical and health outcomes data extracted from a large patient sample drawn from a racially and socioeconomically diverse population receiving care at multiple medical centers over 5 seasons. In addition, the widespread inpatient use of a sensitive multiplex PCR to detect RSV and influenza allowed for the possible detection of a wide spectrum of both diseases [6]. Moreover, since these test results were not immediately available, initial medical management decisions, including hospital admission and treatment decisions, were made based on severity of clinical symptoms rather than virologic diagnosis, minimizing bias resulting from differential admission or management plans for different infections.

## CONCLUSIONS

RSV is a cause of serious illness among hospitalized older adults with morbidity and mortality that may be even more substantial than that caused by influenza, with possibly greater impact on long-term survival. The projected continued increase in the proportion of older adults in the population [25] underscores the growing unmet medical and public health need to develop vaccines and therapeutics directed against RSV. Increased appreciation among adult providers of the frequency and severity of RSV disease among adults will be important to the uptake of RSV vaccines and antivirals after they are introduced.

## Notes

***Financial support.*** The work was funded by Novavax, Inc., Gaithersburg, MD.

***Acknowledgments.*** The authors acknowledge Songyue Chen for his contribution to programming. The authors thank the patients of Kaiser Permanente for helping us improve care through the use of information collected via our electronic health record systems.

***Potential conflicts of interest.*** V. S. is an employee of Novavax and is a Novavax shareholder. H. F. T., L. S. S., B. A., C. F., J. S., Y. L., V.S., and Z. S. received research support from Novavax for this study. H. F. T., L. S. S., B. A., C. F., J. S., Y. L., and Z. S. received research support from Novartis and GlaxoSmithKline for studies unrelated to this research. L. S. S., B. A., J. S., and Z. S. received research support from Dynavax for studies unrelated to this research. H. F. T. and Y. L received research grants from the Centers for Disease Control and Prevention unrelated to this research. All authors have submitted the ICMJE Form for Disclosure of Potential Conflicts of Interest. Conflicts that the editors consider relevant to the content of the manuscript have been disclosed.
